# Cognitive behavioural therapy versus supportive therapy for persistent positive symptoms in psychotic disorders: The POSITIVE Study, a multicenter, prospective, single-blind, randomised controlled clinical trial

**DOI:** 10.1186/1745-6215-11-123

**Published:** 2010-12-29

**Authors:** Stefan Klingberg, Andreas Wittorf, Christoph Meisner, Wolfgang Wölwer, Georg Wiedemann, Jutta Herrlich, Andreas Bechdolf, Bernhard W Müller, Gudrun Sartory, Michael Wagner, Tilo Kircher, Hans-Helmut König, Corinna Engel, Gerhard Buchkremer

**Affiliations:** 1 Department of Psychiatry and Psychotherapy, University of Tuebingen, Germany; 2 Institute for Medical Biometry, University of Tuebingen, Germany; 3 Department of Psychiatry and Psychotherapy, University of Düsseldorf, Germany; 4 Department of Psychiatry and Psychotherapy, University of Frankfurt/Main, Germany; 5 Clinical Center Fulda, Clinic for Psychiatry and Psychotherapy, Fulda, Germany; 6 Department of Psychiatry and Psychotherapy, University of Cologne, Germany; 7 Department of Psychiatry and Psychotherapy, University of Duisburg-Essen, Germany; 8 Department of Clinical Psychology, University of Wuppertal, Germany; 9 Department of Psychiatry and Psychotherapy, University of Bonn, Germany; 10 Department of Psychiatry and Psychotherapy, University of Marburg, Germany; 11 Department of Medical Sociology and Health Economics, Center for Psycho-social Medicine, University Medical Center Hamburg-Eppendorf, Germany

## Abstract

**Background:**

It has been demonstrated that cognitive behavioural therapy (CBT) has a moderate effect on symptom reduction and on general well being of patients suffering from psychosis. However, questions regarding the specific efficacy of CBT, the treatment safety, the cost-effectiveness, and the moderators and mediators of treatment effects are still a major issue. The major objective of this trial is to investigate whether CBT is specifically efficacious in reducing positive symptoms when compared with non-specific supportive therapy (ST) which does not implement CBT-techniques but provides comparable therapeutic attention.

**Methods/Design:**

The POSITIVE study is a multicenter, prospective, single-blind, parallel group, randomised clinical trial, comparing CBT and ST with respect to the efficacy in reducing positive symptoms in psychotic disorders. CBT as well as ST consist of 20 sessions altogether, 165 participants receiving CBT and 165 participants receiving ST. Major methodological aspects of the study are systematic recruitment, explicit inclusion criteria, reliability checks of assessments with control for rater shift, analysis by intention to treat, data management using remote data entry, measures of quality assurance (e.g. on-site monitoring with source data verification, regular query process), advanced statistical analysis, manualized treatment, checks of adherence and competence of therapists.

Research relating the psychotherapy process with outcome, neurobiological research addressing basic questions of delusion formation using fMRI and neuropsychological assessment and treatment research investigating adaptations of CBT for adolescents is combined in this network. Problems of transfer into routine clinical care will be identified and addressed by a project focusing on cost efficiency.

**Discussion:**

This clinical trial is part of efforts to intensify psychotherapy research in the field of psychosis in Germany, to contribute to the international discussion on psychotherapy in psychotic disorders, and to help implement psychotherapy in routine care. Furthermore, the study will allow drawing conclusions about the mediators of treatment effects of CBT of psychotic disorders.

**Trial Registration:**

Current Controlled Trials ISRCTN29242879

## Background

### Positive symptoms and cognitive behavioural therapy

In the last two decades cognitive behavioural therapy (CBT) approaches for patients with schizophrenia have been developed which are specifically designed to reduce severity of positive symptoms. Positive symptoms such as persecutory delusions and hallucinations, which interfere with the patient's ability to maintain social relationships, cause serious distress and life disruption in patients as well as in relatives. They represent hallmark symptoms of psychosis in the schizophrenia spectrum disorders. Even with advances in pharmacological treatments for schizophrenia and other psychotic disorders there is a large subgroup of patients characterised by nonresponse to antipsychotic treatment. Leucht et al. [1] report nonresponse rates between 38% and 76% even when second generation antipsychotic agents are prescribed.

Against this background the investigation of other treatment approaches which may have the potential to reduce positive symptoms has high priority. It has been demonstrated that cognitive behavioural treatment (CBT) has a moderate effect on symptom reduction and on general well being of patients suffering from psychosis. The most recent meta-analyses [2,3] state that the effect size of CBT is .37 for the reduction of positive symptoms. Based on the earlier meta-analysis of Pilling et al. [4] the British National Institute of Clinical Excellence recommended CBT for routine care. In the meantime, also in other countries like Germany [5], CBT is a recommended treatment modality for symptom reduction.

### Open questions

However, many important questions remain unanswered, even if the efficacy of CBT for symptom reduction is increasingly well established.

1. CBT for psychosis has been specifically developed for the reduction of positive symptoms. However, in contrast to more recent meta-analyses the Cochrane meta-analysis of Jones et al. [6] did not find significant reduction of positive symptoms indicating that more large scale clinical trials are needed. Wykes et al. [2] showed a significant heterogeneity in their meta-analysis which might point to unknown moderator effects. In particular the evidence for the specific efficacy of CBT should be improved. When compared to Supportive Treatment, CBT could not as yet demonstrate clear superiority.

2. Patients with psychotic disorders might conceivably experience symptom exacerbation or suicidal crises as a consequence of psychotherapeutic efforts. Negative effects should actively be sought in order to demonstrate safety of the treatment. Therefore, Jones et al. [6] recommended investigating adverse events in CBT trials. However, Tarrier et al. [7] investigated suicidality in CBT-trials and did not find any indication for an increased rate of suicide attempts. More data about treatment safety would be helpful for implementation of CBT, as scepticism among clinicians is widespread.

3. Wykes et al. [2] found that outcome in CBT trials is associated with methodological rigor. Thus, more clinical trials applying rigorous methodology are needed in order to investigate whether treatment effects are stable even in high quality studies.

4. Psychotic disorders, in particular schizophrenia, are causing a considerable economic burden, e.g. in terms of costs of care. As resources for health care a limited, the cost-effectiveness of single health services increasingly gains importance. CBT for psychotic symptoms is currently not available for a majority of patients due to therapeutic scepticism and limited resources. Against this background the question of cost-effectiveness should urgently be addressed.

5. Psychological theories of delusions emphasize either altered attention (selectively attending to evidence in favour of the delusions), or disturbances in making unbiased inferences [8]. There are findings that patients with persecutory delusions preferentially attend threat related stimuli or threat to the subject's self concept [9,10]. Theory-of-mind skills and attributional style together with social perception (i.e. social cue perception and facial affect recognition) are considered as the main sub-processes of social cognition [11]. The evidence in support of these hypotheses is limited and should be increased.

6. The identification of moderators and mediators of treatment effects is a major issue for further development of treatment strategies. However, it has as yet not been studied whether CBT, if successful, alter these biases towards normality [12], and whether the success of CBT critically depends on cognitive and social-cognitive skills. Treatment effects on neurocognitive plasticity have thus far only been described for "cognitive remediation" therapy in one fMRI working memory study with 6 patients. After therapy, a differential activation in the lateral frontal lobe has been demonstrated [13]. Well described cognitive limitations or deficits, e.g. of declarative memory and attention span, arise from enduring (trait) and transient (state) neurobiological alterations in schizophrenia patients, with considerable variation being present between subjects. Such cognitive limitations underlie the development of specific symptoms [e.g. delusions, [14]], are correlated with the degree of insight [15], and are limiting the success of any therapy which, like CBT, is based on verbal learning and requires sufficient attention. Process-outcome research in psychotherapy represents an empirical strategy for determining, which aspects of the therapeutic process are particularly helpful or harmful to patients [16]. This research links the two domains of process and outcome studies. Orlinsky et al. [16] view the therapeutic contract (treatment model, e.g. rational, goal setting, format), the therapeutic operations (therapist interventions), and the therapeutic bond (therapist's and patient's interpersonal behaviour) as the essential factors of the treatment process. These factors were empirically found to be linked with therapy outcome in many psychiatric disorders. However, regarding psychotherapy in psychotic disorders, such findings are still missing up to now. With respect to CBT for positive symptoms in psychotic disorders process-outcome research is not yet a major focus. Studies on this topic focus mainly on effectiveness [e.g. [17-19]].

### Objectives

The major objective of this trial is to investigate whether CBT is specifically efficacious in reducing positive symptoms when compared with non-specific supportive treatment (ST) which does not implement CBT-techniques but provides comparable therapeutic attention.

The trial is accompanied by research addressing (a) process-outcome-relationship dedicated to illuminating the question of the active ingredients in CBT and (b) neurobiological factors of delusion formation using functional magnetic resonance imaging (fMRI) and neuropsychological assessment, as well as (c) treatment research investigating adaptations of CBT in adolescents. Finally (d) problems in relation to disseminating and establishing findings in routine care will be identified and addressed by a project on cost-effectiveness.

## Methods/Design

### General design aspects

This study is a multicenter, prospective, single-blind, parallel group, randomised clinical trial (principal investigator(PI): SK), comparing CBT and ST with respect to the efficacy in reducing positive symptoms in psychotic disorders at nine months after inclusion. The study includes patients with persistent positive symptoms in six study centers applying a systematic recruitment strategy. CBT as well as ST consist of 20 sessions altogether, 165 participants receiving CBT and 165 participants receiving ST (Table 1). The duration of treatment for each study patient will be approximately 36 weeks (i.e., 9 months). The study will be conducted in accordance to GCP and CONSORT. The study has received approval from the local ethics committees and is carried out in accordance with the latest version of the Declaration of Helsinki.

**Table 1:** Study design

Study condition	CBT Cognitive Behavioural Therapy (+ standard care)	ST Supportive Treatment (+ standard care)
Major inclusion criteria	Patients with psychotic disorders, PANSS-Delusion or - Hallucination ≥ 4, symptoms persistent for at least 3 months	

N = 330 (to be included in six study centres)	165	165

Study treatment sessions (treatment duration 9 months)	20	20

Primary Outcome	Positive Symptoms (PANSS) at post treatment assessment (nine months after inclusion)	

post treatment follow-up	24 month	

### Process of recruitment and obtaining written informed consent

The recruitment for this study will address the catchment area of the participating institutions (Departments of Psychiatry and Psychotherapy of the Universities of Bonn (PI: MW), Düsseldorf (PI: WW), Essen (PI: BM and GS), Frankfurt (PI: GW and JH), Köln (PI: AB), and Tübingen (PI: SK) with their associated inpatient and outpatient facilities). It aims at implementing a systematic recruitment plan which will be documented according to CONSORT. For every screened patient the inclusion and exclusion criteria will be recorded. All patients who fulfil the inclusion criteria will be offered to participate in this study. Thus, the resulting sample should represent a geographic cohort.

Under conditions of routine care the patient population addressed by this trial is treated in psychiatric hospitals in case of acute exacerbations, and psychiatric outpatient facilities. In addition, patients are cared for by a considerable number of social psychiatric institutions for supported housing, supported work, and other social psychiatric services.

For the duration of the recruitment phase all inpatients of the participating institutions with psychotic disorders will be screened for their eligibility before discharge from hospital. Information about the study will be provided to the respective patients whenever possible. At the same time a detailed assessment of eligibility will take place. In outpatient departments a complete screening will be conducted for intervals of three months in order to implement comparable strategies for systematic recruitment. In addition, recruitment can also take place in other outpatient services and practices as well as in institutions for supported housing or supported employment. Again, systematic recruitment strategies will be applied.

**Figure 1:**
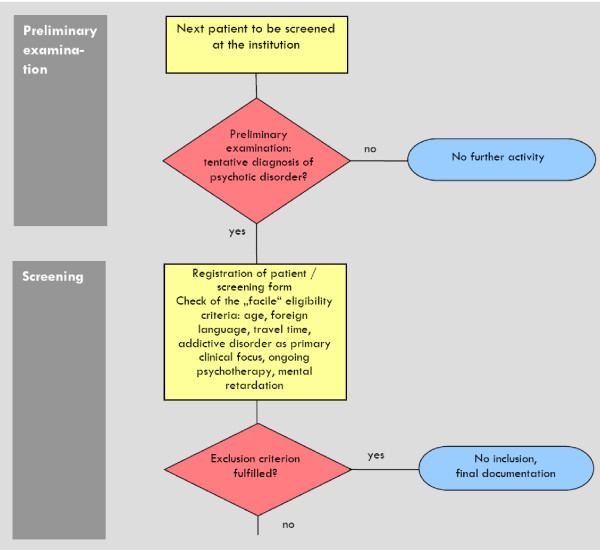
**Process of screening**.

**Figure 2:**
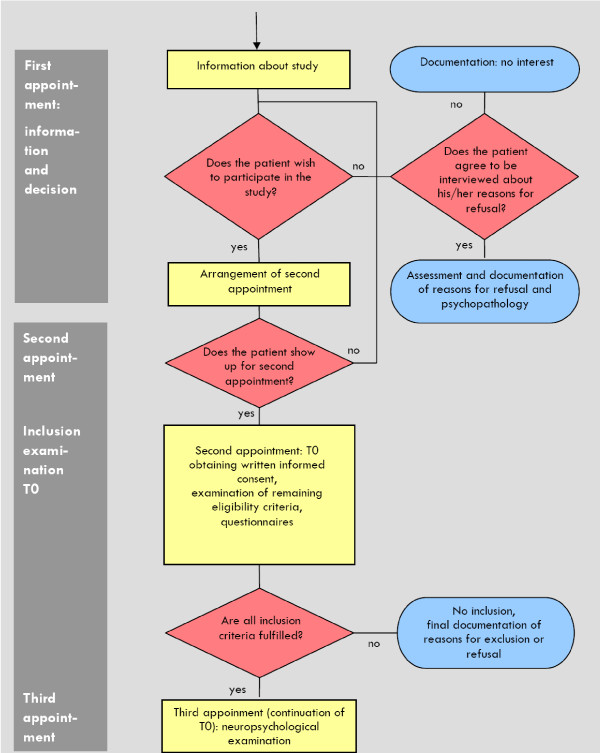
**Informed consent and baseline examination (T0)**.

**Figure 3:**
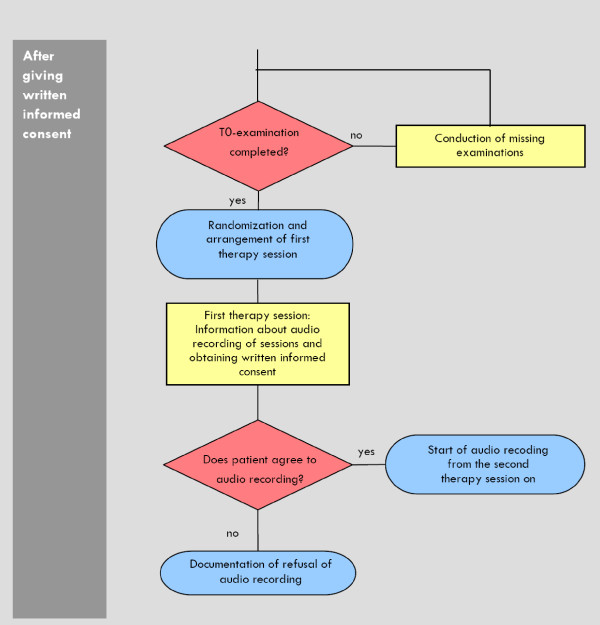
**Randomization and begin of study therapy**.

The registration procedure will be conducted for all patients with a tentative diagnosis of a psychotic disorder (see Figure 1, 2, 3). Patients without "obvious" exclusion criteria (e.g. age, foreign language, living outside of the catchment area, substance dependency as primary problem, mental retardation, ongoing outpatient psychotherapy) will be approached and offered to be informed about the study.

In case of refusal, the patient will be asked whether he is willing to give reasons for refusal and to allow for symptom assessment (PANSS) at the time of refusal. For this purpose a separate patient information and consent form will be applied. Only after giving consent for this interview the reasons for refusal from the viewpoint of the patient and the current symptomatic status will be assessed and recorded. If the patient does not give his consent to be interviewed for his reasons of refusal, the staff member who has provided the information about the trial will give his opinion about the major reason.

In a first appointment potentially eligible patients will be informed about the study using the written patient information. In in-patients study information can be provided in the hospital. The written patient information will be handed out when the patient signifies interest in study participation. In each case, obtaining written consent will take place at the beginning of a second appointment prior to inclusion examination. This second appointment has to take place in an out-patient setting in order to ensure the patient's willingness to start outpatient treatment. If patients have by law a care giver responsible to provide support for the patient regarding health related decisions, patients can only be included in the trial if both the patient and the care giver give their informed consent. The care giver will be asked to show the staff member responsible for inclusion his/her certificate of appointment.

For every screened patient a paper based screening and randomisation form will be filled in and faxed to the Coordination Centre of Clinical Trials (CenTrial). The screening and randomisation form will include a list of questions to be answered during the registration procedure. In particular, the inclusion and exclusion criteria will be recorded. Patient screening and randomisation will only be accepted from authorized investigators. At the end of the screening and randomisation form the investigators will document the intended status of the patient in the trial: (1) as patient to be included and randomised, or (2) as patient not to be included but screened (with or without participation in the refusal survey).

### Inclusion Criteria

In order to be included into the study, patients have to meet diagnostic criteria of schizophrenia (DSM-IV 295.1, 295.2, 295.3, 295.6, 295.9), schizophreniform disorder (DSM IV, 295.4), schizoaffective disorder (DSM-IV 295.7), or delusional disorder (DSM IV 297.1), confirmed by a structured clinical interview (SCID-I). Essential for inclusion is moderate or severe symptom intensity, i.e., a score of 4 or more, on the PANSS-items "Delusions" (P1) or "Hallucinations" (P3). Furthermore, the presence of positive symptoms for at least three months with or without compliance regarding antipsychotic medication is necessary. Other inclusion criteria are fluency regarding the German language, age between 18 and 59, a verbal IQ > 80 assessed by a multiple-choice vocabulary test ("Mehrfachwahl Wortschatz Intelligenz-Test", MWT-B, [20]), and willingness to give informed consent. Criteria for exclusion are any kind of organic brain diseases (other than schizophrenia) according to standard patient examination procedures and diagnosis of substance abuse or substance dependence according to DSM-IV/SCID-I as primary clinical problem.

### Randomization

All patients who give consent for participation and who fulfil the inclusion criteria will be randomized. Randomisation will be requested by the staff member responsible for recruitment and clinical interviews from CenTrial.

In return, CenTrial will send an answer form to the study therapist who is not involved in assessing outcome of the study. This form will include a randomisation number. In every centre closed envelopes with printed randomisation numbers on it are available. For every randomisation number the corresponding code for the therapy group of the randomisation list will be found in inside the envelopes. The therapist will open the envelope and will find the treatment condition to be conducted in this patient. The therapist then gives the information about treatment allocation to the patient. Staff responsible for recruitment and symptom ratings is not allowed to receive information about the group allocation.

As part of the efforts of quality assurance the correctness of the randomisation procedure in each patient will be monitored at the regular on-site visits.

The allocation sequence will be generated by the Institute for Medical Biometry (IMB) applying a permuted block design with random blocks stratified by study centre and medication compliance (favourable vs. unfavourable). The blocks should provide comparable numbers of patients in both conditions at any time in the course of the study. The block size will be concealed until the primary endpoint will be analysed. Throughout the study, the randomisation will be conducted by CenTrial in order to keep the data management and the statistician blind against the study condition as long as the data bank is open. The randomisation list remains with CenTrial for the whole duration of the study. Thus, randomisation will be conducted without any influence of the principal investigators, raters or therapists.

### Blinding

This study implements a single blind design by completely separating treatment and assessment.

Therapists will not be involved in assessing the treatment outcome. Raters will not be allowed to hold treatment sessions. Patients will be informed about their treatment allocation by the therapist but not by the raters. Only therapists will receive information about group allocation. Discussions about study patients are not allowed between raters and therapists. These principles were part of the staff training.

At the beginning of every visit, the raters will instruct the patient not to reveal their treatment condition and not to talk about details of their treatment. The raters will have to complete a "blindness protocol" after each visit. Any unintentional disclosure of the treatment condition will be documented. Further, the raters are asked to guess the study condition of the patient after each assessment. Among all documented guesses the rate of correct guesses should not be significantly different from chance (i.e. 50%).

In order to avoid any bias in data analyses data will be primarily analysed by the intention-to-treat principle. Further, statistical analyses will be conducted by an independent statistician of the Institute for Medical Biometry (IMB). The statistician is not involved in randomisation. The group variable (treatment allocation) will not be included until all data checks are completed. Even in case of severe adverse events no unblinding of raters will be necessary. In these cases therapists will start the appropriate crisis management strategies. If the rater is the first staff member to detect an adverse event he will give notice to the therapist who will implement all appropriate measures.

### Cognitive behavioural therapy

CBT for the treatment of positive symptoms in psychotic disorders is based on general CBT principles. Participants are regarded as active, self responsible individuals. During all phases of the treatment patients are requested to actively participate in the treatment and to take responsibility for decisions how to proceed together with the therapist. The therapeutic process rests on the cooperation between patient and therapist. Whenever necessary, the therapist modifies his intervention in order to help the patient to engage in the therapeutic process.

Treatment is built on a case formulation: Patients and therapist will engage in developing a shared definition of the major problem of the patient. When providing information about psychosis therapists will use a normalising and non-stigmatising style of explanations. The formulation has to address (explicitly or implicitly) persistent positive symptoms. A shared formulation is thought to be a necessary prerequisite for a successful treatment. The specific problems to be addressed in CBT are delusions and hallucinations. The treatment is aimed at helping the patient cope with these symptoms. A major principle of CBT is to link behaviour, emotion and cognition in order to provide a detailed understanding of the patient's problems. Psychotic symptoms are understood as result of dysfunctional ways of perceiving and interpreting social situations. CBT aims at correcting the person's misperceptions, irrational beliefs and reasoning biases as well as at reducing the distress caused by symptoms and the improvement of social functioning. Participants engage in monitoring own thoughts, feelings and behaviours. They are encouraged to test alternative ways of coping with the target symptom. Strategies for the treatment of delusions and delusional processing of hallucinations are to review the information processing (perception bias, jumping to conclusions, attributional bias, theory of mind deficit), to engage in schema work in order to modify potentially delusion related self schemata, to plan activities for reality testing which will provide evidence for or against the delusional conviction, and to help patients reduce the disruption of life and daily activities caused by the delusions. Strategies to reduce hallucinations are to improve the patients coping strategies (e.g. systematic distraction strategies), and to identify and change social or internal stimuli related with increased hallucinatory experiences.

Major stages of CBT can be described as follows:

• Engagement (strategies to foster motivation for treatment participation)

• Assessment (regarding symptoms and social problems)

• Developing understanding of psychotic symptoms using a "normalising" style of providing information )

• Case formulation and treatment planning

• Specific techniques designed to address delusions and hallucinations

• Specific techniques designed to address dysfunctional beliefs and schemata

• Specific techniques designed to improve social functioning

(See Figure 4)

**Figure 4:**
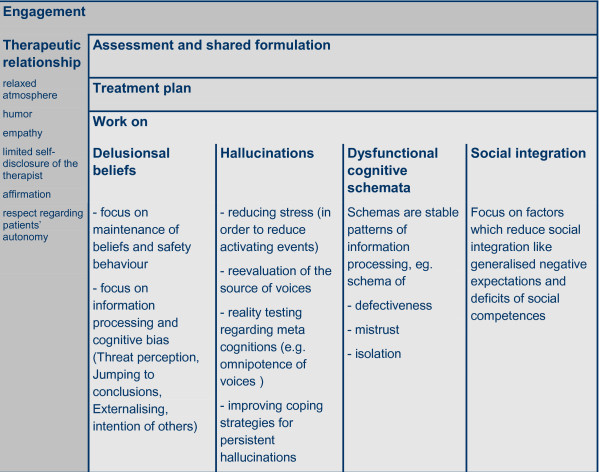
**Cognitive Behavioural Therapy - Overview**.

The CBT manual of the POSITIVE study is published in German language at the research network's homepage http://www.psychose-psychotherapieforschung.de

### Supportive Therapy

ST will be used as comparator in order to control for non-specific elements of therapeutic contact. Psychotherapy outcome is generally thought of as consisting of both specific and non-specific effects. Non-specific effects like emotional support, therapeutic attention, empathic listening, implementation of therapeutic optimism and others are the result of every successful therapeutic relationship. In contrast, therapeutic outcome, which is directly linked to specific and well-defined treatment strategies, is called specific effect. It is hypothesised that CBT produces specific and non-specific factors whereas ST should only result in non-specific factors. ST does not rely on specific theories or assumptions about the causes of positive symptoms in psychotic disorders. ST will focus on the patients' experiences and daily activities. The sessions will focus on neutral topics, such as hobbies, sports, and current affairs. Therapists will engage in listening to the patient, in being empathic, in helping the patient structure the available time and discussing problems in way friends would do. Thus, ST is thought as an active treatment with respect to the patient-therapist relationship and with respect to therapeutic commitment [21]. In the treatment of patients suffering from psychotic disorders these ingredients are viewed to be essential as it has been shown consistently that the social network of these patients is limited. To have at least one trustworthy person to talk to may be the most important ingredient in any kind of treatment. However, with respect to specific processes related to modification of psychotic beliefs, ST is not an active treatment. Strategies specifically designed to change misperceptions or reasoning biases are not part of ST.

Major aspects of ST will be:

• Engagement

• Assessment of social problems and interests of the patient

• Treatment planning

• Focus on housing, work, leisure time, hobbies, and events, as adequate.

**Figure 5:**
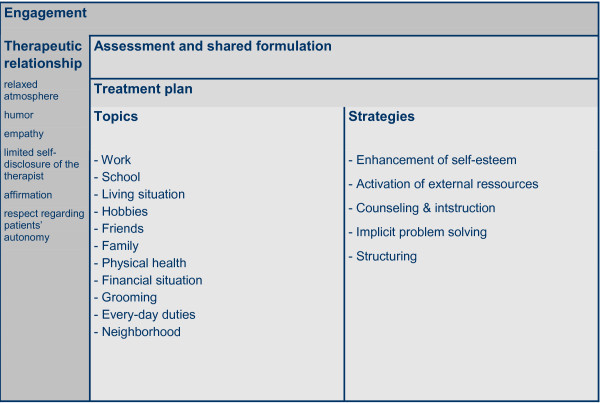
**Supportive Therapy - Overview**.

Psychotic or affective symptoms will not directly be tackled in any way. (See Figure 5)

The ST manual of the POSITIVE study is also published at the research network's homepage http://www.psychose-psychotherapieforschung.de.

### Formal characteristics of study therapies

CBT and ST are individual outpatient treatments of 20 sessions over 9 months (7 sessions in the first seven weeks, followed by 13 fortnightly sessions)

CBT as well as ST will be conducted by specifically trained psychotherapists on the basis of a treatment manual. Each therapist conducts both the CBT and the ST treatment. In order to take responsibility as study psychotherapist, staff members have to have at least one year of clinical experience as clinical psychologist or resident in psychiatry. In addition, they have to be enrolled in formal training programs for cognitive behavioural therapy or have to have completed their formal training.

In the case of interruptions of the study therapies due to rehospitalisation of patients, holidays, other illnesses of patients, illness of therapists etc. the treatment will be continued as soon as possible. However, continuation is only possible within a time frame of 9 months after study inclusion. Thus, within 9 months after study inclusion interruptions of participation will not result in termination of the study treatment, exclusion from the study or a new screening. When continuing the treatment the remaining sessions will be scheduled on a weekly basis as long as necessary. Thus, even patients with longer interruptions will have the chance of getting a maximum of the 20 sessions. In each case, study therapies have to be terminated 9 months after study inclusion (primary endpoint).

### Primary Endpoint

The primary endpoint for the biometrical evaluation of the therapy is the Positive Score of the Positive and Negative Syndrome Scale (PANSS, [22-24]). This psychopathological rating scale represents a common standard rating used in a wide range of outcome studies in schizophrenia. It will be assessed 9 months after inclusion of the participant.

The PANSS positive score is defined by seven items of the PANSS (P1 - P7), all 7-point rated with higher scores representing increasing levels of psychopathology. It will be computed as the mean of these seven items in order to provide a score that is easy to interpret on the scale of a single item.

The German version of the PANSS was evaluated for interrater reliability by Müller et al. [25] and was used for the assessment of the primary endpoint by most of the studies of the German Research Network on Schizophrenia [26].

For the purpose of this trial PANSS is regarded as a validated assessment instrument for positive symptoms. No own validation studies on the PANSS will be conducted. Moreover, the normal distribution and other prerequisites for parametric analyses (e.g. interval scale type) of the PANSS positive-syndrome score will be assumed.

In order to ensure the interrater reliability of the rater responsible for patient assessments in this study, the raters will be trained for PANSS-rating. At the end of the training the interrater reliability between the raters will be assessed using videotaped interviews. Three videos will be assessed by all raters and the reliability will be statistically evaluated by the intraclass correlation coefficient (ICC). The reliability will be regarded as acceptable if the ICC of the PANSS-positive score will be .8 or higher. If the ICC is lower than .8 the rater training will be continued until the criterion is fulfilled. Assessment for reliability will be repeated in case of any change of raters.

During the course of the study the interrater reliability will be assessed regularly every year in order to analyse rater shift. It will be reported whether raters demonstrate a tendency of higher or lower ratings at the end of the study.

As persistent positive symptoms are chronic symptoms which change very slowly a two-year follow-up after completion of treatment will be conducted. The primary endpoint will be analysed also at the end of the follow-up period.

### Endpoints of Safety

Endpoints of safety are

• Death caused by suicide

• Suicide attempt

• Suicidal crisis (explicit plan for serious suicidal activity without suicide attempt) as defined in Calgary Depression Rating Scale for Schizophrenia [CDSS; [27]], item 8, rating 2)

• Severe symptomatic exacerbation, defined by the Clinical Global Impression Scale (CGI) which includes ratings of illness severity, changes in overall clinical status, and therapeutic effects. A rating of CGI2 ≥ 6 and CGI 1≥ 6 would be regarded as severe adverse event.

Information about these safety parameters is recorded in the CRF every four weeks by the study rater as part of the regular clinical assessment.

### Secondary Endpoints

A complete overview over the endpoints of this trial is given in Table 2. Secondary endpoints will cover additional aspects of outcome, such as the course of symptoms and insight into the disorder. Results regarding secondary outcomes will only be interpreted as exploratorily. In particular, PSYRATS [28] provides information about the distress caused by symptoms, AMDP [29] provides additional information about the content of delusions, and SUMD [30] allows for a more detailed analysis of the patient's self awareness regarding symptoms, i.e. insight.

**Table 2:** Timing of assessments of endpoints for safety and efficacy

	T0	T1,2	T3	T4,5	T6	T7,8	T9	F1	F2	F3	F4
	**pre**	**treatment phase** **(monthly assessments)**					**post**	**follow-up** **(every 6 months)**			

**Month**	**0**	**1,2**	**3**	**4,5**	**6**	**7,8**	**9**	**15**	**21**	**27**	**33**

***Inclusion Assessment***											

Structured Clinical Interview for DSM-IV	x										

Anamnestic interview	x										

***Safety***											

Severe Adverse Event asessment^1^		x	x	x	x	x	x	x	x	x	x

CDSS [27]	x	x	x	x	x	x	x	x	x	x	x

Clincal Global Impression (CGI)		x	x	x	x	x	x	x	x	x	x

***Efficacy***											

PANSS [22]	x	x	x	x	x	x	x	x	x	x	x

PSYRATS [28]	x	x	x	x	x	x	x	x	x	x	x

GAF and Social Status	x	x	x	x	x	x	x	x	x	x	x

Blindness Protocol	x	x	x	x	x	x	x	x	x	x	x

CSSRI^3 ^[61]	x		x		x		x	x	x	x	x

EQ-5D [64]	x		x		x		x	x	x	x	x

SUMD [30]	x						x		x		x

AMDP-psychosis items^2 ^[29]	x						x		x		x

Side effect rating scale [65]	x						x		x		

SCL-90-R [31]	x						x		x		x

FSKN [33]	x						x		x		x

Neuropsychological test battery	x						x				

^1^Suicide, Suicide attempts, ^2 ^items 33-58, ^3^includes assessment of medication and medication compliance; CDSS: Calgary Depression Scale for Schizophrenia; PANSS: Positive and Negative Syndrome Scale; PSYRATS: Psychotic Symptom Rating Scales; GAF: Global Assessment of Functioning; CSSRI: Client Sociodemographic and Service Receipt Inventory; EQ-5D: Euro Quality of Life - 5 Dimensions; SUMD: Scale to Assess Unawareness of Mental Disorder; SCL: Symptom Check List; FSKN: Frankfurter Selbstkonzeptskalen (Frankfurt Self-Concept Scales)

Treatment effects in the field of psychiatric disorders should always be assessed also from the viewpoint of the patient. However, a disorder of self evaluation is part of the positive syndrome which is the reason for choosing observer ratings as primary endpoint. On the other hand, the general rating of patients about their symptoms is important and is represented by the following secondary endpoint.

The Symptom Checklist [SCL-90-R; [31]] assesses subjectively felt impairments due to somatic and psychic symptoms within a frame of seven days. The SCL-90-R for example also assesses psychotic or depressive symptoms. Thus, self ratings of these symptom dimensions seem to be a sensible completion of symptom ratings. As CBT is supposed to reduce symptoms we hypothesize, that SCL-scores will be lower in CBT compared to ST at T9.

Low self-esteem might be viewed as a product of the schizophrenic patient's experience of positive as well as negative symptoms and its deleterious social consequences [32]. On this background, the self concepts as measured by the Frankfurt Self-Concept Scales [FSKN, [33]] will be assessed. The subscales of this self rating questionnaire assess attitudes towards the own person (e.g. self esteem). Cognitive behavioural therapy might have a beneficial impact on self-esteem in schizophrenic patients [32]. Since self concepts do usually change slowly the FSKN will only be applied at baseline, post-treatment, 6-month follow-up, and 12-month follow-up.

Traditional instruments that measure self-esteem may not relate directly to the schema construct as outlined in recent cognitive models. The Brief Core Schema Scales [BCSS; [34]] aim to provide a theoretically coherent self-report assessment of schemata concerning self and others in psychosis. The scales assess four dimensions of self and other evaluation: negative-self, positive-self, negative-other, positive-other. The BCSS will be applied at the same assessment points as the FSKN.

### Quality control/Monitoring

As an instrument for quality control and quality assurance the clinical trial will be monitored. Monitoring will be performed by the Coordination Centre for Clinical Trials at University Hospital Tuebingen (CenTrial) according existing standard operating procedures (SOPs). The purposes of the monitoring are to verify that the rights and well-being of the subjects are protected, that the reported trial data are accurate, complete, and verifiable from source documents, and that the conduct of the trial is in compliance with the study protocol/amendments, with GCP, and with applicable regulatory requirements. The extent and nature of the monitoring will be determined in a monitoring manual before starting the trial. For every on-site visit a monitoring report will be submitted to the sponsor. Status reports of the monitor will inform the sponsor regularly about the actual status in the trial sites.

### Data Management

Case report forms must be completed according to the following schedule:

a) Before the treatment starts: the patient must be screened/randomised at CenTrial. For that purpose all relevant data must be reported.

b) Documentation of the treatment and follow-up visits: Each visit should be documented immediately.

c) Upon occurrence of a Severe Adverse Event (SAE)

All SAEs occurring during the observation period of 9 months must be reported by fax to the sponsor's medical expert, the medical director of the Department of Psychiatry and Psychotherapy of the University of Tuebingen. All forms must be dated and signed by the responsible investigator or one of his/her authorized staff members.

This study is designed to be documented mainly via internet. The study software *koordobas*, an Oracle-based application of the IMB, will be used for the data management. This application was in full compliance with GCP-requirements (e.g. audit trail, validation) at the start of the trial. The eCRF data are reported by authorized investigators via internet on the specific case report forms (eCRFs). The case report forms (eCRFs) must be completed, dated and signed electronically by the investigator or one of his/her authorized staff members as soon as the requested information is available. The list of staff members authorized to sign case report forms (with a sample of their signature) have been sent to CenTrial by the responsible investigators before the start of the study.

In all cases, it remains the responsibility of the investigator to check that case report forms are completely and correctly filled in. The data manager will perform extensive consistency checks on the eCRFs and issue Query Forms in case of inconsistent data. Those Query Forms must be immediately answered and signed by the investigator (or an authorized staff member). The original must be returned to CenTrial and a copy must be appended to the investigator's copy of the eCRFs.

If an investigator (or an authorized staff member) needs to modify an eCRF after the original eCRF has been filled in and returned, he/she can change it by notifying the Data Centre electronically appending a print out of the notification to his own copy of the eCRFs. All modifications will be protocolled by the audit trail of the study software.

All study related data (electronic as well as on paper) will be stored for 10 years in the archive of the Department of Psychiatry and Psychotherapy, University of Tuebingen.

The audio tapes of therapy sessions will be stored in locked filing cabinets. They are to be labeled with the patients ID only. Access to the audio tapes is permitted only with written permission of the principal investigator. The audio tapes will be destroyed after finishing the data analysis.

Assessment, storing, processing, and deleting of person related data will be conducted in accordance to German law.

### Sample size calculation

The primary endpoint analysis will be conducted with linear mixed models (LMM, [35]). As software for sample size calculation for the analysis of longitudinal data using multilevel mixed models is not available, we calculated the sample size for classical ANOVA using nQuery 4.0.

The power calculation is based on published results about CBT for persistent positive symptoms. For the comparison between CBT and TAU Tarrier et al. [36] reported Effect Sizes (ES: (mean_TAU_-mean_CBT_)/SD_TAU_) of 0.33-0.66 for the 18 month follow-up. Tarrier et al. (1998) found an ES of 0.48, Kuipers et al. [37] an ES of 0.6, and Sensky et al. [19] an ES of 0.5. In a review Gould et al. [38] found a range of ES from 0.2 to 1.26. The reported variance differs to a great extent indicating considerable differences with regard to samples or treatments. A recent effect size analysis applied broader inclusion criteria for studies and resulted in an ES of .57 for acute patients (post-treatment analysis) and an ES of 0.27 in chronic patients [3]. These reviews show considerable efficacy of CBT when compared to treatment as usual. However, this study focuses on the difference between CBT and Supportive Treatment (ST). Unfortunately, the power calculation is more difficult for this comparison as fewer studies are available. According to a review of Tarrier et al. [36] the following studies have included ST-control groups: Tarrier et al. [18], Haddock et al. [28], Pinto et al. [39], Lewis et al. [40], and Durham et al. [41]. The effect sizes vary between -.49 in a study including only 21 patients [28] and .99 in a study with 37 patients [39]. In addition, sample characteristics and endpoints are different between the studies. Thus, it does not seem possible to make assumptions about the ES for the comparison between CBT and ST based on the literature.

Regarding drop out rates there is also much heterogeneity with a range between 0% and 36% [36,38]. The majority of studies reports drop out rates of less than 20%. As measures of quality control will be applied and monetary incentives for participation in the follow-up examinations will be offered we expect a drop out rate of about 20%.

On this background we aim to identify an effect size of more than .35 as significant given an anticipated drop out rate of 20%. An ES of .35 would be obtained if the PANSS Scores (Positive Syndrome) at the post treatment assessment were 12 for CBT and 14.5 for ST with a standard deviation of 7.14. An ES of less than .35 would be of limited clinical relevance.

This results in n = 130 per group for a power of 80% and a two-sided significance level of 5% (sample size calculated by nQuery 4.0, Panel MGT0). The confirmatory statistical analysis will be based on the intention-to-treat principle. Patients with missing PANSS-scores at T9 (post-treatment) will be included with the last observation carried forward (LOCF). In case of missing PANSS-scores at T9 the treatment effect will presumably be underestimated by using LOCF. To compensate for this underestimation the sample size should be adapted for drop out. Thus, we plan to include 330 patients (165 per study condition).

As assumptions about the real effect size cannot be based upon the literature we calculated different scenarios: in case of a lower effect size and/or drop out of more than 20% the statistical power will be reduced. For example, a reduced ES of 0.2 would result in a power of only 36% for the drop out of 20%. An increased drop out of 30%would reduce the power to 74% for the minimum ES of .35. On the other hand, a more favourable ES of 0.45 would increase the power to 85% for the maximum drop-out rate of 20%.

With a sample size of 330 individuals (165 each therapy group), ten assessments per patient, one primary analysis variable (therapy) and one covariable (center), the power should also be sufficient for a Mixed Model [42].

Table 3 gives an overview over the number of patients required in the different stages of the trial and the required effort for treatment and assessment. In order to successfully include 330 patients 6 study centers have been included which were committed to participate actively in this trial.

**Table 3:** number of patients required in the different stages of the trial

	total	per center
required number of eligible patients (with 75% refusal)	1304	217
number of patients to be included (incl. 20% drop out)	330	55
number of patients to be analysed (ITT, LOCF)	330	55
number of patients to be analysed per protocol	260	44
treatment sessions (CBT and ST)	5530	922
number of visits	4620	770

### Statistical Analysis

The primary endpoint for the biometrical evaluation (responsible statistician: CM) is the PANSS-Positive-Score of the Positive and Negative Syndrome Scale at the time of 9 months after inclusion. It will be analysed after completion of T9-assessments.

The statistical hypothesis for the confirmatory test of the primary endpoint is that the treatment groups are significantly different when analysed using multilevel linear mixed models with treatment and study centres as levels of analysis and adjustment for baseline values. The decision for maintaining or rejecting the null hypothesis will be made applying a two-sided test with α = 0.05. A two-sided test will be chosen as the published results about the comparison of CBT and ST are inconsistent. The observed effects will be described by use of means including the appropriate (one-sided) 95%-confidence intervals.

The confirmatory statistical evaluation of the efficacy of the CBT in this trial will be restricted to the primary endpoint. Only the rejection of the null hypothesis will be interpreted as statistical evidence for the efficacy of CBT. The confirmatory statistical analysis will be based on the intention-to-treat principle (ITT). In addition, a "per protocol" (PP) analysis will be conducted

All secondary endpoints will be compared and statistically assessed for descriptive purposes and not in a confirmatory sense. The aim of the analysis is explorative data analysis, not hypothesis testing or generation of evidence for efficacy. Because of the explorative character of this part of the analysis, no a priori statistical analysis plan exists. If adequate, secondary endpoints will be compared and statistically assessed using covariance techniques with baseline values and centers as covariates. Changes of scores over time will be modeled using linear or non-linear or nonparametric models as adequate. In addition, appropriate statistical methods of explorative data analysis including graphical methods and descriptive statistics will be used. No interim analysis and no subgroup analyses are planned.

### Medication

Regarding psychopharmacological treatment the study is open and requires no restriction of treatment. The sample size of this trials justifies the expectation of equal distribution of type (classical vs. atypical antipsychotics), dose, rate of non-adherence to medication, prescription of other psychopharmacological treatment (antidepressants, mood stabilizers, benzodiazepines) in both treatment groups.

However, both the medication dose and the medication compliance are potential confounders which have to be controlled for. In particular, it could be the case that medication will not be completely independent from the study condition. As medication has the potential to influence the course of symptoms it is important to observe the medication carefully to allow for detailed analysis of this aspect. Medications and doses will be documented monthly. Side effects will be assessed at baseline as well as at the post-treatment assessment T9.

Medication compliance [43] will be rated monthly along a 7-point scale (with 1 = total rejection of medication, and 7 = active cooperation and full acceptance of medication).

### Assessment of adherence to treatment manuals

To evaluate the adherence to the manuals a number of measures will be assessed. First, in order to systematically assess characteristics of form and content as well as aspects of adherence of treatment therapists filled in structured session reports after each treatment session. The session reports give information whether the treatment session was conducted as scheduled or cancelled by the patient and whether the session began on time or delayed. Further, the session reports show the duration of the session, the primary and secondary foci of intervention (e.g. establishing treatment goals, work on delusions), the use of manualized treatment material, reasons for non-adherence to the agenda (e.g. due to symptomatic worsening, focus on current problems, non-compliance of patient or other reasons), accomplishment of homework by the patient, and the cooperation of the patient as rated by the therapist. The cooperation rating scale is a fully anchored ordinal scale with 1 = excellent, 2 = adequate, 3 = sufficient, and 4 = poor. The session reports will be available for all sessions conducted. An individual study therapy will be considered as having been conducted according to manual if a patient has attended at least 14 treatment sessions. Further, two thirds of the sessions conducted have to fulfil the following criteria: duration >40 and <60 minutes, use of manualized treatment materials or strategies, and at least sufficient cooperation of the patient.

Second, all treatment sessions (CBT and ST condition) will be audio taped if patients give their consent. A maximum of four audio tapes of each patient will randomly be selected for analyses: one of the first 3 sessions (early phase), one of the sessions 4-10 (early middle phase), one of the sessions 11-17 (late middle phase), and one of the last 3 sessions (late phase). This procedure is independent of the actual number of treatment session a patient has participated in. The audio tapes will be checked with regard to manual adherence. Guided by a checklist it will be assessed whether or not the treatment session followed the CBT or the ST manual with regard to content, the material worked on, and the formal characteristics (e.g. duration of the session). The ST checklist also comprises items regarding an irregular application of CBT-specific techniques. Our adherence checklists will be subjected to analysis of interrater reliability. Since mistrust and paranoia are core symptoms of chronic psychotic disorders, we expect a rate considerably below 100% of patients who give their written informed consent for audio taping their therapy.

### Assessment of unspecific mechanisms of action

According to Orlinsky's and colleagues' review [16] the strongest evidence linking process to outcome concerns the therapeutic alliance. Therapeutic alliance proved to be a common ingredient of all psychotherapeutic interventions [44] and to be at least modestly correlated with outcome [45,46]. The therapeutic alliance as well as other unspecific mechanisms of action (e.g. clarification, activation of resources, emotional involvement, problem solving) will be assessed using the Bernese Post Session Reports [47] for patients (BPSR-P) and therapists (BPSR-T). In the framework of the German Research Network on Schizophrenia we conducted factor analyses of both instruments in a sample of 111 first episode patients with schizophrenia participating in CBT for relapse prevention. We identified dimensions relating to the therapeutic alliance, therapy progress, emotional involvement, dissatisfaction with therapy, and others. Both instruments will be applied at the end of each therapy session of the CBT and the ST condition. The continuous application of the session reports will allow examining the course of the above mentioned unspecific therapeutic mechanisms during the CBT and ST. We expect no differential course of these unspecific factors between the two treatment conditions.

### Assessment of therapist's competence

Establishing empirical collaboration between the patient and the therapist as a means for cognitive restructuring is a major aspect of the therapists' competence in CBT. To measure the extent of empirical collaboration the four randomly selected audio tapes will be analysed by means of the Cognitive Therapy Scale for Psychosis [CTS-Psy; [48]]. The CTS-Psy consists of two subscales each with five items. The items are rated on the original nominal scale (1 = appropriately included, 0 = inappropriately omitted, and 9 = appropriately omitted) as wells as on a 5-point interval scale where higher scores indicate better competency. We designed this 5-point Likert-scale to prevent potential ceiling effects. Scale I "general skills" includes the items agenda setting, feedback, understanding, interpersonal effectiveness, and collaboration; scale II "specific skills" covers guided discovery, focus on key cognitions, choice of intervention, homework, and quality of intervention. The specific skills will be measured only in the CBT condition. The CTS-Psy demonstrates excellent inter-rater reliability, good validity, and sensitivity to changes [48]. Furthermore, we will conduct own analyses on the interrater reliablity of our German adaptation of the CTS-Psy.

Regarding the application of general skills we expect no difference between the CBT and the ST condition. Further, according to the rationale of CBT, we hypothesize that the quality of a therapist's specific skills correlates with therapeutic outcome (symptom reduction) in the CBT condition.

The research addressing treatment adherence, the unspecific mechanisms of action, as well as the therapist's competence are integrated within the sub-project "Process and outcome in CBT for positive symptoms in psychotic disorders" (PI: AW).

### Assessment of safety of interventions during the treatment phase

Safety of psychological intervention in schizophrenia has been assessed in terms of suicide rates and rates of serious symptom deteriorations. The Cochrane Reviews on Family Intervention [49], on Cognitive Behavioural Treatment of Positive Symptoms [50], on Cognitive Remediation [51], and on Psychoeducation [52] could demonstrate that neither the suicide rate nor the rate of relapse was increased in psychological intervention. The same result was reported by Tarrier et al [7] who conducted the most subtle analysis yet on this topic and included also an ST condition in their study. Thus, there is no indication in the literature for an increased risk for patients as a consequence of their participation in the treatment described in this protocol. However, based on clinical experience it seems important to consider the following major risks:

(a) Increase of positive symptoms as a consequence of therapeutic overstimulation: It is known that positive symptoms may increase if the psychosocial stress is greater than the coping ability of a patient. Thus, a forced therapeutic approach may represent stress for a patient increasing the risk for symptom exacerbation.

(b) Suicide, suicide attempt or suicidal crisis could occur as a consequence of dysfunctional coping with the negative psychological and social consequences of the disorder.

However, this study provides optimal conditions to prevent major risks, to detect symptom exacerbations early, and to intervene early and sufficiently. (a) The symptoms are assessed frequently in short intervals by the rater as well as by the study therapist. (b) Therapists are trained to react to symptom deterioration. They will adapt their strategy to the patients' needs. This is completely compatible with the treatment manual. (c) The therapist will discuss the treatment strategy with clinically experienced supervisors. (d) A psychiatrist responsible for routine care is involved in the treatment. Independent of the study this psychiatrist will initiate crisis intervention whenever required.

As described above there is no indication for specific risks or enhanced rates of adverse events as a consequence of participation in CBT or ST. However, as the safety of interventions for positive symptoms in schizophrenia have not been studied extensively this study will control for severe symptom exacerbations and suicidal crises as adverse events.

Severe adverse events to be observed at every assessment throughout the trial are:

• Death caused by suicide

• Suicide attempt

• Suicidal crisis (explicit plan for serious suicidal activity without suicide attempt) as defined in Calgary Depression Rating Scale for Schizophrenia [CDSS; [27]], item 8, rating 2)

• Severe symptomatic exacerbation, defined by the Clinical Global Impression Scale (CGI) which includes ratings of illness severity, changes in overall clinical status, and therapeutic effects. A rating of CGI2 ≥ 6 and CGI 1≥ 6 would be regarded as severe adverse event.

The safety parameters are part of the regular clinical follow-up examination conducted by the study rater every four weeks. In addition, study participants remain in their usual routine outpatient treatment and will therefore see their independent psychiatrist regularly. The routine care psychiatrist and the study therapist are requested to exchange information about the status of the patient and to provide an optimal individual treatment. The study rater will communicate any information about adverse events to the routine care psychiatrist and the study therapist. Thus, the study provides an optimal framework to immediately detect any complication in the treatment.

Information about the safety parameters is recorded in the CRF every four weeks by the study rater. In addition, therapists are requested to assess the safety parameters in every session and to record their assessment in the session protocol.

The observation of these events should result in a statistical remarkable result, if the incidence of events in one of the two groups will be higher than in the other group. The statistical observation will be done for a significance level of α = 0.2 (two sided) and a power of 0.8 with the help of the software PEST (distributed by Whitehead).

Analyses will be conducted three times: after 100, 200 and 300 patients reaching T3. When analysing the safety data, the full observation period of all patients and all available data will be considered. The safety analyses will be conducted by a statistician (CE) not involved in the design and analysis of this trial, using coded group labels A and B blinded for the real therapy groups.

The results will be reported directly to the members of the independent advisory board. In case of rejection of the null hypothesis of equal incidence rates in the two groups, the advisory board will decide whether or not the study has to be stopped.

We do not expect any difference regarding illness related events between the groups.

### Time schedule of the study and duration of subject participation

Table 4 gives an overview over major phases of this trial. A long preparation phase was necessary due to the multicenter design and the requirements of a publicly funded clinical trial. The recruitment phase was completed in January 2010. The recruitment phase had to be extended as the availability of patients who where willing to give consent was limited in some study centers. However, the designed sample size could be included and the post-treatment assessments (T9) will be finalized in October 2010. Analyses of major outcome will begin in November 2010. Long-term follow-up data will be collected until October 2012.

**Table 4:** Study phases

Phase	Time
Grant application and preparation	March 2005 - March 2007
Recruitment	April 2007 - January 2010
Treatment completion and completion of post treatment assessment	February 2010 - October 2010
Follow-up until 24 months after treatment	November 2010 - October 2012
Start of Analysis of primary outcomes	November 2010

A single patient participates for a duration of 33 months consisting of the treatment phase (9 month) and the follow-up phase (24 months). The time of "first patient in" until "last patient out" will be 67 months.

### Funding, role of funders, and "sponsor" responsibilities

This study is publicly funded by the German Federal Ministry of Education and Research (Bundesministerium für Bildung und Forschung, BMBF), project number 01GV0618. The study is part of the BMBF research program "Research Networks on Psychotherapy". The funding agency selected projects on the basis of the vote of an international review board. It does not exert any influence during the trial. The responsibilities of the "Sponsor" in terms of the guidelines of good clinical practice in clinical trials (ICH-GCP, E6) has been taken by the University Hospital of the University of Tübingen which delegated responsibility to the head of department of Psychiatry and Psychotherapy.

## Associated research

### Cognitive deficits and biases

For the assessment of neurocognitive deficits, a short and reliable neuropsychological battery consisting of tests measuring verbal intelligence, attention, executive functions, and memory is administered. This battery has been widely employed in the BMBF funded German Research Network on Schizophrenia [53] and a substantial database on the relationship of cognitive deficits with psychopathological symptoms, course of illness, and response to therapy has been gathered [54]. The assessment of cognitive biases or cognitive styles is an emerging field of research, and several experimental studies have found evidence of specifically altered performance in delusional subjects [55,56]. Therefore, the sub-processes of social cognition which might serve as mediating mechanisms will be assessed in detail in the sub-project "Cognitive deficits and cognitive biases underlying delusional symptoms and therapeutic change" (PI: MW). Attribution style is assessed with the Internal, Personal and Situational Attribution Questionnaire [IPSAQ; [57]]. Usually, the attributional style of paranoid patients is altered in that they, similar to depressed patients, make global and stable explanations for negative events, but, unlike depressives, they preferentially assume external causes, and particularly other people to be responsible [8]. Another aspect of disturbed inferential thinking relates to the evaluation of hypotheses. In tasks requiring one to make a good guess based on prior evidence, paranoid patients jump to conclusions prematurely, as if they need less evidence to be sure (this style has been termed epistemological impulsivity). One method to assess such a reasoning style is the Beads in a Jar Task of Garety and colleagues [58]; an adapted computer version of this task [59] is employed here. Finally, standardized pictures from the Pictures of Facial Affect set [PFA; [60]] are applied to assess accuracy and speed of facial affect recognition.

### Neural correlates of cognitive biases

A functional magnetic resonance imaging (fMRI) study (PI: TK) investigates major mediating factors of Blackwood's model. Using fMRI differential effects of CBT and ST on cerebral activation is being investigated. The present study investigates neurophysiological processes underlying the development and amelioration of symptoms of delusion. The neural correlates of jumping to conclusions and attributional bias will be investigated. The paradigms of the fMRI study will be complementary to those applied in a larger sample via neuropsychological experiments outside the scanner. Extensive reliability and quality control measurements ensure the validity of data across different centres. The following questions will be addressed: What are the neural correlates of delusions taped by the dysfunctional processes of attributional bias and jumping to conclusions? Can future therapeutic success be predicted on the basis of specific brain activation patterns already before treatment? Which components of neural circuits can potentially by altered by CBT? Are there distinct brain structures that can be linked to delusions?

### Cost effectiveness

An associated project assesses the cost effectiveness of the treatment (PI: HHK). The direct and indirect costs of both study arms are calculated prior, during and after therapy using a modified version of the „Client Sociodemographic and Service Receipt Inventory" [61]. The CSSRI assesses the overall resource utilization of patients as well as productivity losses. To estimate costs these quantities are then valued with market prices. When market prices are not available administrative prices or mean costs are used to estimate so called "shadow prices".

To assess the health effects of CBT and ST, two different measures are used. On the one hand the EQ-5D [62], a generic measure of subjective health related quality of life comprising a health profile and a visual analogue scale is used prior, during and after therapy. From the EQ-5 D, health state utilities are derived to calculate quality adjusted life years (QALYs) [63]. On the other hand an objective measure of positive symptoms (PANSS-Score) is used to quantify treatment response.

A Markov model is built to estimate the ICER using long term costs and effects beyond the time frame of the study. Markov models simulate the course of a disease over time and thus allows for calculating long term costs and effects.

### Adaptation of CBT for adolescents

This sub-project (principal investigator: AB) represents a pilot study which focuses on the evaluation of extensions of CBT for adolescents with early onset psychosis (EOP). Objectives are to develop a modified CBT (mCBT) for adolescents with EOP, to explore its acceptance and feasibility and to provide data for a realistic estimation of achievable effect size. The study is a multicenter, prospective, parallel group, randomised controlled trial. Forty-two patients will be recruited. All participants will receive individual optimised psychiatric treatment as usual (TAU). mCBT will be provided for 50% of the patients (n = 21) in addition to TAU. mCBT is an outpatient treatment which consists of 20 individual sessions in nine months and five psychoeducation sessions with parents. All sessions will be conducted by specifically trained psychotherapists on the basis of a treatment manual.

The primary endpoint will be the positive syndrome of the PANSS at T9 (post treatment assessment). Monthly assessments during the treatment phase will closely monitor the course of symptoms. Patients have to fulfil DSM-IV criteria of schizophrenia, schizoaffective disorder, or delusional disorder, confirmed by a structured clinical interview (SCID-I). Decisive for inclusion is a score of 4 or more on the PANSS-items "Delusions" (P1) or "Hallucinations" (P3) or „Unusual thought content" (G9) representing a moderate or severe symptom intensity. Furthermore the presence of positive symptoms for at least four weeks or more is necessary.

## Discussion

### Relevance of the POSITIVE study

This clinical trial is part of efforts to intensify psychotherapy research in the field of psychosis in Germany, to contribute to the international discussion on psychotherapy in psychotic disorders, and to help implement psychotherapy in routine care. Furthermore, the study will allow drawing conclusions about the mediators of treatment effects of CBT of psychotic disorders.

In an innovative approach the network combines clinical trials on the efficacy of CBT with research designed to analyse active ingredients of the treatment. The POSITIVE Study will presumably be the largest full scale clinical trial comparing CBT with ST. This comparison will allow drawing conclusions about the specific efficacy of CBT for the treatment of persistent positive symptoms in psychotic disorders. The projects of the research network will give information about the processes of CBT, the effects of CBT on cognitive biases as well as the neural basis of theses biases. The latter have not yet been studied, in particular with respect to treatment outcome. Further, a full economic evaluation of CBT will be conducted. To this day, no such data on cost effectiveness are available yet.

### Strength of the POSITIVE study design

There are several strengths of our study design. To summarize, within a multicenter design a systematic recruitment procedure with clear inclusion criteria is implemented. Randomisation is applied independent of investigators and therapists. The study is single blind and the success of blinding will be assessed a posteriori.

Reliability checks for the primary endpoint have been conducted prior to the first patient inclusion and thereafter once a year. Thus, we will be able to control for intra-rater shift over time.

Further, assessment and analysis of severe adverse events is a crucial component of the POSITIVE Study. Thus, our clinical trial will be the first which gives detailed information about the safety of CBT on persistent positive symptoms.

As an instrument for quality control and quality assurance the clinical trial will be monitored by a Coordination Centre for Clinical Trials. Manualized treatments with predefined adherence checks, regular supervision, and process-outcome analyses assure the quality of the study therapies.

Finally, the POSITIVE study provides an adequate sample size for the expected moderate treatment effect, has a predefined primary endpoint and multilevel secondary endpoints. The elaborated statistical analysis will be done by an external statistician.

### Lines of interpretation

There are two main lines of interpretation of the results of POSITIVE study.

First, according to our hypothesis, CBT might be superior to ST with regard to the reduction of positive symptoms. This result will be interpreted as an evidence for the specific efficacy of CBT. In this case it will be interesting, whether the cognitive biases (e.g. jumping to conclusions, external attribution style) have been changed and normalized in the CBT only and whether these changes can also be observed on a neural level. A significant association of the change of the cognitive biases on the one hand side and the change in positive symptoms on the other hand side would support the basic assumption of CBT approaches that the cognitive biases are factors which actually mediate the treatment effect. In addition such a result would substantially support psychological models of delusion formation [12] as it would show that psychological processes are involved not only in the development but also in the reduction of positive symptoms. However, if changes in positive symptoms are not associated with changes in biases questions will arise regarding the hypothesised mechanism of action in CBT. The health economical analysis will add an additional aspect of evaluation as it will focus on cost effectiveness and not on efficacy.

Second, CBT and ST might show no significant difference regarding reduction of positive symptoms. In this case CBT has no specific effect on positive symptoms and symptom changes are independent of the investigated psychotherapeutic treatments. As this trial does not include a "treatment as usual" (TAU) condition in order to maximise the statistical power the question will remain open, whether CBT and ST had any effect on positive symptoms. Effects of the "natural" course and effects of medication can not be identified using the present design. Even, if CBT and ST lead to comparable changes in positive symptoms it will be important to analyses changes in cognitive biases as these treatments might build on different mechanisms of action.

## List of abbreviations used

BMBF: Bundesministerium für Bildung und Forschung (German Federal Ministry of Education and Research); CBT: Cognitive Behavioral Therapy; CDSS: Calgary Depression Rating Scale for Schizophrenia; CGI: Clinical Global Impression; CONSORT: Consolidated Standards of Reporting Trials; CTS-Psy: Cognitive Therapy Scale for Psychosis; DGPPN: Deutsche Gesellschaft für Psychiatrie, Psychotherapie und Nervenheilkunde (German Psychiatric Association); DSM-IV: Diagnostic and Statistical Manual of Mental Disorders, Forth Edition; DSMC: Data and safety monitoring committee; eCRF: electronic Case Report Form; TAU: Treatment As Usual; FSKN: Frankfurt Self-Concept Scales; IMB: Institute for Medical Biometry; IQ: Intelligence Quotient; mCBT: modified Cognitive Behavioral Therapy; MWT-B: Mehrfachwahl Wortschatz Intelligenztest (multiple choice vocabulary intelligence test); PANSS: Positive and Negative Syndrome Scale; SCID-I: Structured Clinical Interview for the DSM-IV, Axis I; ST: Supportive Therapy

## Competing interests

The authors declare that they have no competing interests relating to the content of the manuscript.

## Authors' contributions

SK is the principal investigator and grant holder of the POSITIVE study and is - together with GB speaker of the POSITIVE research network. SK, AW, and GB participated in the design of the study. AB, BM, GS, MW, GW, JH, WW and SK are the local principal investigators. Further, AW, AB, TK, HHK, and MW are the principal investigators and grant holders of the associated research. CM and CE are the statisticians of the study. All authors read and approved the final manuscript.
